# A Case of Rupture of the Intestines, Produced by a Fall from a Horse

**Published:** 1851-10

**Authors:** P. D. McCulloch

**Affiliations:** Murfreesborough, Tenn.


### SELECTIONS

FROM

### FOREIGN AND HOME MEDICAL JOURNALS.

---

*A Case of Rupture of the Intestines, produced by a fall from a horse—resulting in Death.* By P. D. McCulloch, M. D., of Murfreesborough, Tenn.—This case occurred with a negro boy belonging to Dr. Avent, aged 9 years.

He was thrown from a horse on Saturday, April the 27th, 1850, between 3 and 5 o'clock in the afternoon.

Dr. Avent being absent, I was called to see him at 2 o'clock, Sunday morning. I found him complaining of much pain over the whole abdomen, but when directed to put his hand on the part that gave him so much pain, he would invariably put it over the region of the Pyloric extremity of the stomach. He was in a state of partial collapse. He vomited up his dinner, which he had taken but a short time before the accident, and continued to retch for several hours after vomiting, but did not throw up any matter of any kind.

Under the free use of external stimuli, the circulation was sufficiently re-established to justify me in the use of the lancet. I took a small quantity of blood from his arm, but soon found that he would not bear its loss. I therefore checked the bleeding, and re-applied the sinapisms to the extremities and abdomen. I made a very minute examination of the whole body, but could detect no lesion of either the soft or bony parts.

I gave him at 5 o'clock in the morning seven grains of calomel, one grain of ipecac, the sixth of a grain of morphia, and directed that his bowels should be moved at 12 o'clock by castor oil, or an enema. At the time I left him, his pulse was 100 to the minute and tolerably full, which was six o'clock in the morning.

Dr. Avent reached the patient at 8 o'clock, and shortly after he commenced sinking. Dr. Avent used different internal stimuli, together with sinapisms to the extremities, all of which proved abortive, as the patient continued to sink till 3 o'clock in the afternoon of Sunday, at which time he expired. His abdomen became very much swollen previous to death. Dr. Avent informed me that when pressure was made upon the abdomen it produced tetanic twitching of the muscles of the right shoulder and arm.

Autopsy 19 hours after death.

On laying open the abdominal cavity, a great quantity of very offensive gas escaped. There was also a large quantity of effused fluid. There were traces of intense inflammation of the whole peritoneum, but more especially that portion lining the walls of the cavity. Having a suspicion that there might be a rupture of the intestines, we commenced tracing them from the pylorus. We soon found the contents of the bowel, together with about half a dozen large intestinal worms, floating loose in the cavity of the abdomen. On tracing the bowel on, we found that the rupture had occurred in the jejunum, six feet from the pylorus, and in the opposite side to the main seat of pain, as complained of by the patient. There were two ruptures, about an inch apart, and in opposite sides of the bowel. Between the ruptures there were several dark gangrenous patches. There was no lesion in any of the other viscera. The place where the accident occurred was perfectly level.—*Proceedings Med. Soc. Ten.*

